# Reply to letter to the editor

**DOI:** 10.1016/j.jdin.2021.01.006

**Published:** 2021-03-02

**Authors:** Sweta Hasmukh Rambhia, Kinjal Deepak Rambhia

**Affiliations:** a Just Care Dental and Skin Clinic, Mumbai, India; b Department of Dermatology HBT Medical College and R N Cooper Municipal General Hospital, Mumbai, India

*To the Editor:* We thank Arbache et al.^1^ for their interest in our recent article. We concur with the author's opinion regarding the potential for improved precision and depth control using tattoo devices as compared to handheld needles. We also agree that it may be easier to perform and that treatment time is shorter, especially for patients having multiple lesions.^2^^,^^3^

However, the use of tattoo devices may also theoretically lead to the inadvertent delivery of higher concentrations of 5-fluorouracil, an antimetabolite agent, thereby increasing the risk of local and systemic side effects. The settings of the tattooing needle and its depth may need to be changed according to skin thickness. The benefits of using a hypodermic needle are that it can be easily manipulated based on the thickness of the skin of the treatment area and the presence of pinpoint bleeding. We recommend the application of topical anesthesia or local anesthesia for increased patient comfort.

Procedures should always be performed under aseptic conditions when tattoo devices, microneedling devices, or a simple hypodermic needle are used for treatment. As mentioned in our article, the use of a hypodermic needle for the treatment of idiopathic guttate hypomelanosis is a simple, cost-effective, office-based procedure that can be easily performed in remote areas or in resource-poor settings that have no access to tattoo machines and consumables.

## Conflicts of interest

None disclosed.
